# Einthoven Dissertation Prizes 2023

**DOI:** 10.1007/s12471-023-01803-1

**Published:** 2023-08-15

**Authors:** J. J. Piek

**Affiliations:** Department of Cardiology, Heart Centre, Amsterdam University Medical Centres, location Academic Medical Centre, University of Amsterdam, Amsterdam, The Netherlands

The dissertation prize is named after Willem Einthoven, a pioneer in cardiovascular medicine who recorded the first human electrocardiogram (ECG) in 1902, for which he was awarded the Nobel Prize in 1924. The annual Einthoven Dissertation Prize is an initiative of the Netherlands Heart Institute (NHI) and the Netherlands Society of Cardiology (NVVC) to select the top three cardiovascular theses published in the year 2022. The jury received a total of 15 PhD dissertations for selection. The ranking of the theses was based upon a combination of parameters which included the curriculum vitae of the candidate, the scientific originality of the PhD thesis and its relevance for the cardiovascular field. Moreover, several objective bibliometric parameters were used including the number of articles in citation index journals, both in PubMed and the Web of Science (WOS), the number of citations in WOS, the Hirsch Index, and finally the contributions of the candidate as first author. Based upon this evaluation, the jury selected the following nominees: Aernoud Fiolet (University Medical Centre Utrecht), Michiel Henkens (Maastricht University Medical Centre) and Jorrit Lemkes (Amsterdam University Medical Centre). The members of the jury were Professor Pieter A.F.M. Doevendans (NHI), Dr. Joan G. Meeder (NVVC), Dr. Marco J.W. Götte (Cardiovasculair Onderwijs Instituut), Dr. Martin E.W. Hemels (Werkgroep Cardiologische Centra Nederland) and Dr. Eric Dubois (President *Concilium Cardiologicum*). The three candidates presented their PhD theses at the DCVA-NLHI Translational Cardiovasacular Research Meeting. We congratulate the laureates for their excellent scientific work and their presentations during the meeting.

## Summary: Colchicine in coronary disease

Historically, innovation in medical research is often two-faced: an indefatigable aim to improve care at the inevitable expense of increased costs. For that reasons, contemporary science should focus on how to re-*use the available to enable the more sustainable*. The aim of this thesis was, therefore, also two-sided: to investigate how to repurpose a low-cost drug to treat a high-cost disease, and how to conduct high-fidelity clinical research in a very efficient way.

Coronary disease is a lipid-driven inflammatory disease. We have shown that a crucial part of the inflammatory pathway in atherosclerosis is caused by deposition of cholesterol crystals in the fibroatheroma. This process is incited via inflammasome proteins and also seen in other crystal-related maladies, such as gout. Highly specific human monoclonal antibodies (e.g. canakinumab) can target these pathways, but are expensive and not widely available. We investigated colchicine, a low-cost drug with a well-known safety profile, to dampen relevant inflammatory mediators. We have shown that short- and long-term colchicine treatment lowers typical inflammatory biomarkers such as C‑reactive protein and interleukin‑6, but also lowers more complex biochemical routes, such as neutrophil activity and extracellular inflammasome protein levels.

Using this biological principle, we designed an international clinical trial in which patients with chronic coronary syndromes were randomised to low-dose colchicine (0.5 mg once daily) or placebo. We recruited at outpatient clinics, without any preselection on inflammatory profile and irrespective of the baseline cardiovascular risk. The primary outcome was cardiovascular death, myocardial infarction, ischaemic stroke, or ischaemia-driven coronary revascularisation. The trial was conducted in 30 Dutch and 13 Australian hospitals. The trial was investigator-initiated and sponsored by the Netherlands Organisation for Health Research and Development (ZonMW). By employing risk-based monitoring strategies, innovative trial medication dispensing and following large in-kind contributions from the participating cardiology centres, the costs of the trial were kept to less than a tenth of equivalent industry-sponsored trials.

After randomising 5522 patients and a median follow-up of 29 months, we demonstrated a 31% relative risk reduction in the occurrence of the primary outcome in patients allocated to colchicine as compared with placebo. This effect size was similar in women and men, and irrespective of the time to the last coronary event. The number needed to treat with colchicine was equal to or lower than intensive lipid lowering, extensive blood pressure lowering or prolonged dual antiplatelet therapy. The results of the main trial and the ancillary papers led to a treatment recommendation with low-dose colchicine in the 2021 European Society of Cardiology guidelines for secondary prevention of cardiovascular disease. When evaluating a patient with coronary disease in the current era, we suggest that after lifestyle modification the four medical pillars of treatment that a cardiologist should consider are anti-thrombotic, anti-lipidaemic, anti-hypertensive and anti-inflammatory.

In another effort to improve the conduct of sustainable research, we showed that routinely collected healthcare data from electronic healthcare records can efficiently and accurately be used to identify eligible patients for trial participation. In addition, rather than using patient interviews by research staff, we have shown that baseline and outcome data can be collected accurately using this method, with almost identical results to those found using conventional manual data collection.

The research described in this thesis has contributed to the confirmation of the anti-inflammatory hypothesis in the treatment of coronary disease, changed guideline-recommended therapy and provided important guidance for durable execution of clinical trials. In summary, by using the *available, we enabled the sustainabl*e, and improved the prognosis of patients with coronary disease, while demonstrating cost-effective innovation.

*Aernoud T.L. Fiolet*

Department of Cardiology, University Medical Centre Utrecht, Utrecht, The Netherlands

A.T.L.Fiolet@umcutrecht.nl

## Summary: Improving diagnosis and risk stratification of cardiomyopathies across the ejection fraction spectrum—The past, present and future

Daily, 80 individuals become hospitalised and 20 die due to heart failure (HF) in the Netherlands. In total, around 250,000 people are diagnosed with HF in our country, accompanied by care-related costs that already exceed 800 million euros yearly. Worldwide the prevalence of HF even exceeds 38 million patients. This prevalence is expected to increase further during the upcoming years due to the ageing population and the growing occurrence of other HF-related risk factors, such as diabetes mellitus and obesity.

Future HF-related research should address the challenges in early detection, prevention and management of HF and cardiomyopathies to reduce the societal, economic and healthcare impact of this debilitating syndrome. In-depth characterisation of HF patients using registry-based research will be an essential asset to accomplish this. When conducting registry-based studies, a researcher faces a wide variety of logistic hurdles, which often limit the number of subjects or amount of data included in these studies. These hurdles include but are not limited to the fact that routine clinical data often needs to be collected manually before it can be used for research purposes and the follow-up of these patients and collection of additional data (e.g., to determine quality of life or evaluate the cost-effectiveness of certain treatments) is time-consuming. These hurdles often hold back the performance of in-depth cardiomyopathy or HF research across the entire spectrum of the left ventricular ejection fraction (LVEF), which is regrettable since categorising HF based on LVEF results in an enormous oversimplification of this complex syndrome.

Large-scale registries with real-world data will play a pivotal role in moving the current HF field forward and boost the efficacy of studies such as those presented in the first part of the thesis. These registries will form the foundation for multidisciplinary data, and hypothesis-driven (multi-omic) approaches that can challenge LVEF as the cornerstone of HF classification. HF registries, including unselected subjects, will provide real-world insights into clinical practice, prognosis and temporal trends, and expose novel therapeutic targets which can be subsequently challenged in (registry-based) clinical trials.

Over the past years, the Maastricht Cardiomyopathy Registry team created a future-proof foundation for a multidisciplinary (early) cardiomyopathy and HF registry (the mCMP-registry; as presented in the second part of the thesis). The logistic hurdles faced by the mCMP-registry study team during this time were tackled to improve the way HF registry-based research is conducted. This ultimately resulting in the launching of the mCMP-registry in 2021. The scalability of the mCMP-registry logistics allows other centres to easily join this initiative. The mCMP-registry will allow researchers to perform research more efficiently and enable researchers to unravel the complexity of the HF syndrome beyond the currently used HF nomenclature.

*Michiel Henkens*

University Medical Centre Maastricht+, Maastricht, The Netherlands

michiel.henkens@mumc.nl

## Summary: Timing of coronary angiography in acute coronary syndromes: The TRANSIENT and COACT trial

Numerous studies have focused on the optimal invasive strategy in patients with acute coronary syndromes (ACS). The preferred timing of coronary angiography and revascularisation in patients with transient ST-segment elevation myocardial infarction, however, remained unclear. In the multicentre randomised Timing of Revascularisation in Patients with Transient ST-Segment Elevation Myocardial Infarction (TRANSIENT) trial we compared an ST-segment elevation myocardial infarction (STEMI) like approach with a non-ST-segment elevation myocardial infarction (NSTEMI) like approach in 142 patients with this subset of ACS. We found that patients who present with transient STEMI can be treated with both an immediate (STEMI-like) or delayed (NSTEMI-like) invasive strategy with similar outcomes. Our results showed that patients with transient STEMI have a very limited infarct size with a relatively benign outcome, independently of the timing of coronary angiography. This means that logistic considerations such as time of day and availability of the catheterisation laboratory can safely be taken into account when choosing the optimal time for intervention.

The optimal timing of coronary angiography in patients successfully resuscitated from out-of-hospital cardiac arrest (OHCA) without ST-segment elevation on the ECG was also a matter of debate. The multicentre Coronary Angiography after Cardiac Arrest (COACT) trial randomised 552 of these patients to either an immediate invasive strategy or a delayed invasive strategy with coronary angiography after neurological recovery. We found that in patients who are successfully resuscitated after OHCA, with a shockable rhythm and no signs of STEMI, a strategy of immediate angiography does not improve survival at 90 days or 1 year, nor does it improve left ventricular function compared with a delayed angiography strategy. The focus of treatment in these patients, directly after the cardiac arrest, should therefore be on post-arrest care on the ICU, including target temperature management.

The results of both the TRANSIENT and COACT trials are in line with other studies which investigated the effect of an immediate coronary intervention in patients with ACS without persisting ST-segment elevation on the ECG, and showed no benefit of an immediate invasive strategy. However, a number of trials found that high-risk patients with NSTEMI might benefit from coronary angiography within 24 h compared with a further delayed strategy.

The benefit of an immediate percutaneous coronary intervention (PCI) on outcome in patients with STEMI, however, is well established and is therefore currently the preferred treatment. An important difference between patients with STEMI and those with NSTEMI is the higher rate of occlusion of the culprit coronary artery resulting in decreased myocardial perfusion and ongoing infarction in patients with STEMI. The results of our studies therefore further support the theory that immediate PCI mainly benefits patients in need of reperfusion therapy and less those in whom the culprit vessel is open.

*Jorrit Lemkes*

Amsterdam University Medical Centre, Amsterdam, The Netherlands

j.lemkes@amsterdamumc.nl

Aernoud Fiolet won the first prize, Michiel Henkens the second prize and Jorrit Lemkes the third prize.

